# p53 is required for brain growth but is dispensable for resistance to nutrient restriction during *Drosophila* larval development

**DOI:** 10.1371/journal.pone.0194344

**Published:** 2018-04-05

**Authors:** Esteban G. Contreras, Jimena Sierralta, Alvaro Glavic

**Affiliations:** 1 Biomedical Neuroscience Institute and Department of Neuroscience, Faculty of Medicine, Universidad de Chile, Independencia Santiago-Chile; 2 Center for Genome Regulation, Department of Biology, Faculty of Science, Universidad of Chile, Las Palmeras Nuñoa, Santiago-Chile; CINVESTAV-IPN, MEXICO

## Abstract

**Background:**

Animal growth is influenced by the genetic background and the environmental circumstances. How genes promote growth and coordinate adaptation to nutrient availability is still an open question. p53 is a transcription factor that commands the cellular response to different types of stresses. In adult *Drosophila melanogaster*, p53 regulates the metabolic adaptation to nutrient restriction that supports fly viability. Furthermore, the larval brain is protected from nutrient restriction in a phenomenon called *‘brain sparing’*. Therefore, we hypothesised that p53 may regulate brain growth and show a protective role over brain development under nutrient restriction.

**Results:**

Here, we studied the function of p53 during brain growth in normal conditions and in animals subjected to developmental nutrient restriction. We showed that *p53* loss of function reduced animal growth and larval brain size. Endogenous *p53* was expressed in larval neural stem cells, but its levels and activity were not affected by nutritional stress. Interestingly, *p53* knockdown only in neural stem cells was sufficient to decrease larval brain growth. Finally, we showed that in *p53* mutant larvae under nutrient restriction, the energy storage levels were not altered, and these larvae generated adults with brains of similar size than wild-type animals.

**Conclusions:**

Using genetic approaches, we demonstrate that p53 is required for proper growth of the larval brain. This developmental role of p53 does not have an impact on animal resistance to nutritional stress since brain growth in *p53* mutants under nutrient restriction is similar to control animals.

## Background

Organisms grow and acquire a proper size to ensure their viability. In order to do so, they adapt to the different environmental conditions, maintaining their growth rate during development. Animal growth is determined by the relationship between genetic factors and environmental circumstances. Among the later, changes in food availability and intake force the animal and its cellular metabolism to adjust to the different nutritional conditions, which are constantly challenging animal’s physiology [1]. However, it is not well understood whether the genetic mechanisms that control growth during normal diet, can also facilitate adaptation to nutritional stress.

It has been well described that the function of the central nervous system (CNS) and adult neurogenesis are sensitive to environmental changes such as diet, exercise and drugs [2]. Conditions of undernourishment or overnutrition alter the function of the brain, including learning, memory and the formation of new neurons [3]. Interestingly, the mammalian brain is protected against intrauterine growth restriction produced by food shortage in a phenomenon known as “*brain sparing*”, in which the organism in some way adapts to maximise nutrient and oxygen supply to the brain [4].

Among the genes that could have a role in the response to nutritional stress during development, p53 has been shown to promote the survival of adult flies under nutrient restriction and to modulate glucose and lipid storage consumption [5]. p53 belongs to the p53 family of transcription factors that is also composed by p63 and p73. p53 has been extensively characterised as a tumour suppressor gene and mutant forms of the *p53* locus are found in more than half of human cancers [6, 7]. Hence, *p53* mutant mice are viable, but are predisposed to generate spontaneous tumours [8–10]. Additionally, some *p53* mutant mice die during development due to defects in CNS development [11, 12], showing a role for p53 during normal animal development. p53 is induced and stabilized by different types of cellular stresses, including genotoxic damage, oncogene activation, and nutrient and oxygen deprivation [13]. The response evoked by p53 varies depending on the type of stress and cellular context [14]. For instance, upon low DNA damage, p53 triggers cell cycle arrest, antioxidant response and the activation of the DNA repair machinery promoting cell survival, whilst severe DNA damage produces p53-mediated apoptosis and senescence [15–18]. In cells deprived of nutrients, p53 induces autophagy and modulates glucose and lipid metabolism, activating catabolic pathways to maintain cellular and energy homeostasis [15, 19]. However, p53 regulation of metabolism is highly dependent on the cellular context [14]. For example, in cell competition assays in *Drosophila melanogaster*, p53 enhances glycolytic metabolism, promoting cell survival [20]. On the other hand, p53 can also reduce glycolysis while promotes oxidative phosphorylation by the negative regulation of several enzymes of the glucose metabolism [15, 21].

In *Drosophila melanogaster* there is only one member of the p53 family: Dmp53 or p53 [22–24]. Initially, p53 was described to mediate apoptosis in *Drosophila* tissues [22, 24]. After apoptosis induction, p53 also promotes proliferation in healthy tissue in a compensatory manner [25–27]. Furthermore, p53 regulates growth and proliferation in a cell non-autonomous manner to keep organ proportions, maintaining tissue homeostasis after stress [28]. As in vertebrates, the loss of the *p53* locus generates fertile adult flies without an apparent phenotype, suggesting that p53 is not required during development under normal lab conditions [29, 30]. However, p53 has diverse roles during stress and pathological conditions, for instance *p5*3 mutants are sensitive to radiation and show higher rates of genome instability [29]. In a *Drosophila* model of neurodegeneration, p53 plays a neuroprotective function, preventing apoptosis, and inducing the expression of synaptic genes in the adult brain [31].

Based on the strong evidence showing the protective role of p53 against stress conditions, and particularly on nutritional stress, we analysed the effect of *p53* mutation during *Drosophila* development under normal diet and nutrient restriction. We show here that *p53* loss of function alters pupariation timing and larval growth, delaying larval brain growth. In the larval brain, p53 is expressed in neural stem cells and specific knockdown in this stem cell population reduces brain growth. To understand p53 function under nutritional stress, we used a paradigm in which larval brain continues growing after nutrient restriction [32], resembling the ‘*brain sparing*’ phenomenon seen in humans [4]. We found that *p53* expression and transcriptional activity in neural stem cells in the larval brain is independent of this nutritional condition. Finally, we demonstrate that p53 is not required to maintain brain growth under nutrient restriction, and the metabolism of energy storage in this condition is not affected in *p53* mutant larvae.

## Results

### p53 controls larval growth and development

**Fig 1:**
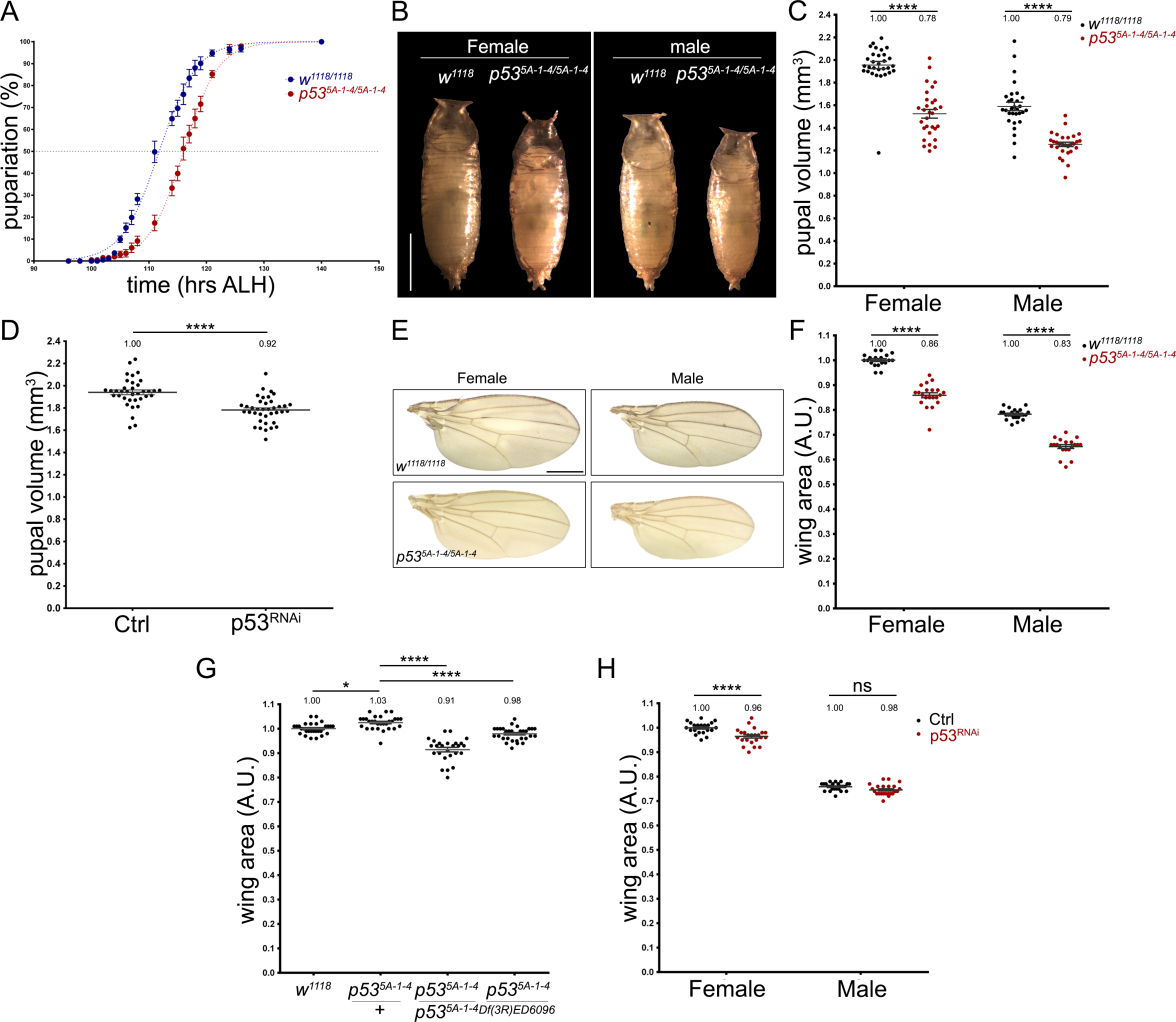
Larval developmental timing and growth are affected by the loss of *p53*. **(A)** Graph showing pupariation curves of wild-type (*w*^*1118/1118*^ in blue) and *p53* null (*p53*^*5A-1-4/5A-1-4*^ in red) larvae. Each point represent the average percentage of pupariation of 5 groups of 37–40 larvae **(B)** Images of female and male pupae from wild-type (*w*^*1118/1118*^) and *p53* null (*p53*^*5A-1-4/5A-1-4*^) genotypes. Scale bar is 1 mm. **(C)** Quantification of wild-type (*w*^*1118/1118*^) and *p53* null (*p53*^*5A-1-4/5A-1-4*^) pupal volume. “n” is 30 pupae for each genetic condition. **(D)** Quantification of pupal volume from *tub-GAL*4 crossed to *w*^*1118*^ (Ctrl) or *UAS-shp53* (p53^RNAi^). “n” is 36 and 38 pupae for Ctrl and p53^RNAi^ condition respectively. **(E)** Wing images of female and male wild-type (*w*^*1118/1118*^) and *p53* null (*p53*^*5A-1-4/5A-1-4*^) flies. Scale bar is 1 mm. **(F)** Quantification of wing area of female and male wild-type (*w*^*1118/1118*^) and *p53* null (*p53*^*5A-1-4/5A-1-4*^) flies. For female *w*^*1118/1118*^ “n” is 18, for male *w*^*1118/1118*^ “n” is 23, for female *p53*^*5A-1-4/5A-1-4*^ “n” is 21 and male *p53*^*5A-1-4/5A-1-4*^ “n” is 20 **(G)** Quantification of wing area of female wild-type (*w*^*1118/1118*^, “n” is 29), female heterozygous *p53* mutant (*p53*^*5A-1-4/+*^, “n” is 29), female homozygous *p53* mutant (*p53*^*5A-1-4/5A-1-4*^, “n” is 26) and female hemizygous (*p53*^*5A-1-4*^*/Df(3R)ED6096*, “n” is 32) flies. **(H)** Quantification of wing area of female and male flies of *tub-GAL4* crossed to control (*w*^*1118*^) or *UAS-shp53* (p53^RNAi^). “n” for female Ctrl is 21, male Ctrl is 24, female p53^RNAi^ is 23 and male p53^RNAi^ is 23. * and **** means p-value < 0.05 and <0.001 respectively. ns is non-significant. Check S1 Table for details of the statistical analysis for each graph.

Given the evidence showing that loss of function of *p53* is partially necessary for mice embryonic development [11, 12], we analysed whether p53 regulates larval development in *Drosophila melanogaster*. Hence, we determined the time of pupariation of both wild-type (*w*^*1118*^) and *p53* null mutant (*p53*^*5A-1-4*^) larvae, observing a delay in *p53* mutant pupariation of approximately 4.2 hrs (Fig 1A). Because puparation timing defects are associated with growth alterations, we analysed pupal size as an indication of larval growth (pupal size reflects the maximum size that a larva reached before pupariation). We observed that *p53* mutant pupae were approximately 20% smaller than wild-type pupae (Fig 1B and 1C). This decrease was also observed when a short-hairpin RNAi against *p53* was constitutively expressed during the entire larval development (Fig 1D), showing that p53 is required for proper larval growth.

To assess whether these defects in larval growth also affect adult tissue size, we quantified the wing area of female and male wild-type and *p53* mutant flies (Fig 1E). We found that the average wing area of *p53* mutant (*p53*^*5A-1-4*^) flies was significantly smaller than control (*w*^*1118*^) flies (Fig 1F). To discard that this phenotype was produced by the genetic background of the *p53*^*5A-1-4*^ mutant stock, we analysed the wing area of wild-type (*w*^*1118*^), heterozygous *p53* mutant (*p53*^*5A-1-4/+*^), homozygous *p53* mutant (*p53*^*5A-1-4/5A-1-4*^) and hemizygous *p53* mutant (*p53*^*5A-1-4*^*/Df(3R)ED6096*). Although heterozygous *p53* mutant wings were slightly, but significantly, bigger than *w*^*1118*^ homozygous controls, wings of both homozygous and hemizygous *p53* mutants were significantly smaller than the wings of heterozygous *p53* mutants (Fig 1G), confirming that p53 is required for proper wing growth. Finally, we constitutively expressed a short-hairpin RNAi against *p53* during the entire *Drosophila* development, finding a significant decrease in the wing area of female adult flies compared to control conditions, but no significant changes were observed in male wing size (Fig 1H). These results support the hypothesis that p53 regulates larval developmental timing and growth, which have consequences that can be seen in the size of the adult wings.

### Larval brain growth is regulated by p53

**Fig 2:**
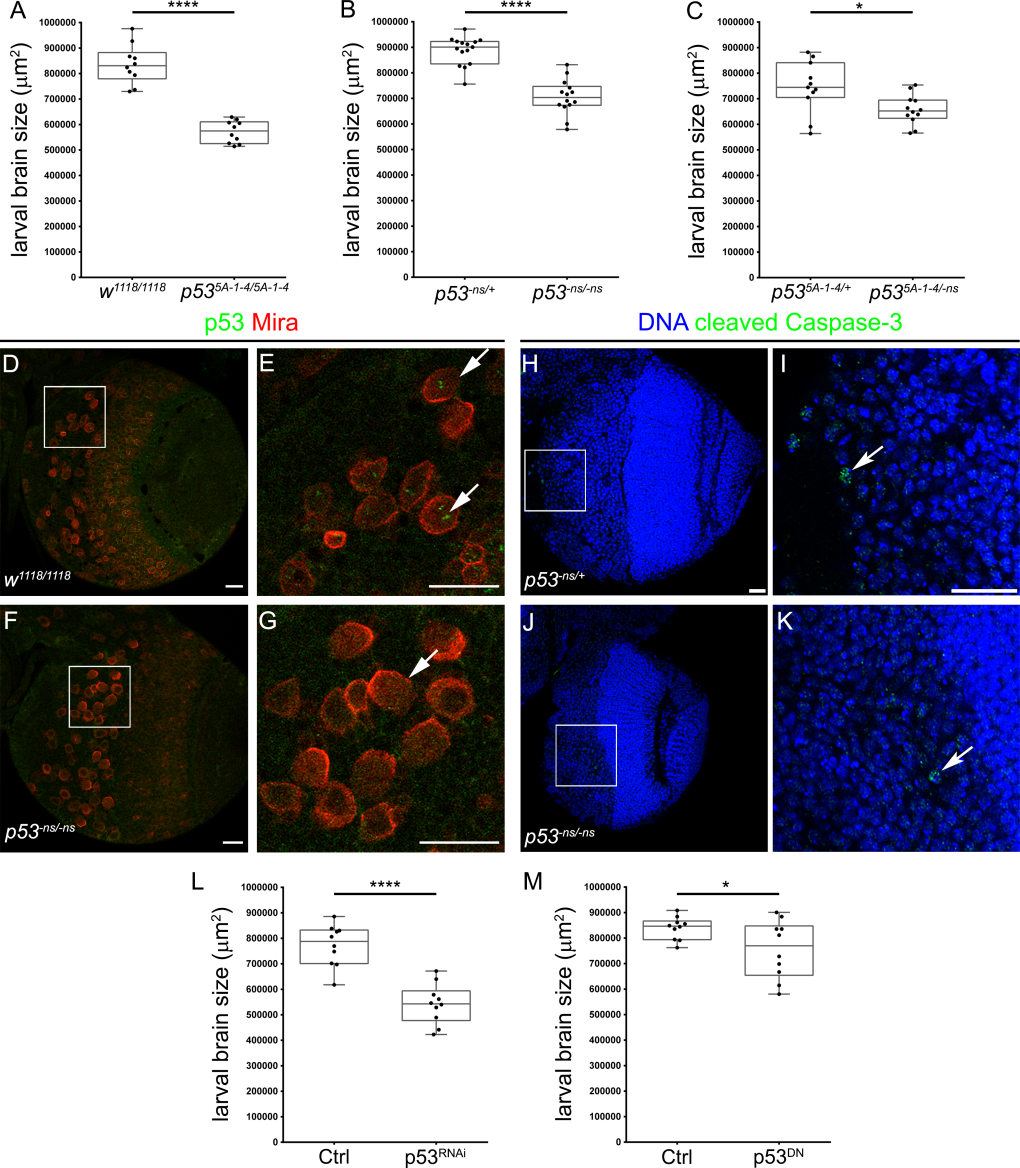
p53 is necessary in neural stem cells for brain growth. **(A-C)** Graphs showing brain size quantifications at 96 hrs ALH. Larval brains from the following genotypes were used: **(A)** wild-type (*w*^*1118/1118*^, “n” = 10) and *p53* null mutant (*p53*^*5A-1-4/5A-1-4*^, “n” = 10), **(B)** heterozygous (*p53*^*-ns/+*^, “n” = 15) and *p53* null mutant (*p53*^*-ns/-ns*^, “n” = 14), **(C)** heterozygous (*p53*^*5-1-4/+*^, “n” = 11) and *p53* null mutant (*p53*^*5-1-4/-ns*^, “n” = 12). **(D-G)** Immunostaining of **(D, E)** wild-type (*w*^*1118/1118*^) and **(F, G)** *p53* mutant (*p53*^*-ns/-ns*^) brains using antibodies against p53 (green) and Miranda (Mira, in red). Arrows point to neural stem cells. Scale bars are 20 μm. **(H-K)** Immunostaining of **(H, I)** heterozygous (*p53*^*-ns/+*^) and **(J, K)** *p53* mutant (*p53*^*-ns/-ns*^) brains using antibody against cleaved Caspase-3 (green) and DNA stained with DAPI (bue). Arrows point to apoptotic cells. Scale bars are 20 μm. **(L)** Graphs showing *pros-GAL4* crossed to control (*w*^*1118*^, “n” = 10) or *UAS-p53*^*R155H*^ (p53^DN^, “n” = 10), and **(M)** *insc-GAL4* crossed to control (*w*^*1118*^, “n” = 10) or *UAS-shp53* (p53^RNAi^, “n” = 10). Unpaired t-tests were used in all experiments. * and **** means p-value < 0.05 and <0.001 respectively.

Since our results show that p53 controls larval growth, we analysed changes in brain size in wild-type and *p53* mutant larvae. We found that the brain of third instar wild-type (*w*^*1118*^) larvae were significantly bigger than homozygous *p53* mutant (*p53*^*5A-1-4*^) brains (Fig 2A). We confirmed this decrease in larval brain growth using a different null mutant allele (*p53*^*-ns*^ null allele) (Fig 2B) and the heteroallelic combination (*p53*^*5A-1-4/-ns*^) (Fig 2C). These results indicate that p53 is necessary for larval CNS development.

Given the role of p53 during larval brain growth, we analysed p53 expression pattern in the larval brain. We assessed p53 protein levels in third instar larval brains by immunofluorescence, finding that p53 staining was mainly present in cells positive for the neural stem cell marker Miranda (Mira) (see arrows in Fig 2D and 2E). This staining was completely lost in *p53* mutant (*p53*^*-ns*^) brains (Fig 2F and 2G), confirming that the signal was specific to p53. Thus, this selective expression in neural stem cells suggests that p53 is involved in the regulation of neurogenesis in larval brains.

Because *p53* is expressed in other larval tissues in addition to neural stem cells, we specifically studied p53 function in neural stem cells using the GAL4/UAS system [33]. Thus, we used the neural stem cell specific drivers: *pros-GAL4* and *insc-GAL4* to express a dominant-negative form of p53 (p53^DN^) or a short-hairpin RNAi (p53^RNAi^). Using both genetic tools, we observed a significant decrease in brains size compared to control animals (Fig 2L and 2M). These results suggest that p53 is necessary in neural stem cells for proper brain growth during larval development.

In order to understand how loss of function of *p53* generated smaller larval brains, we checked the levels of apoptosis in larval brains. Analysing the levels of an apoptotic marker (cleaved Caspase-3, Fig 2H–2K), we found similar low levels of apoptosis in larval brains for both genetic conditions, heterozygous control (*p53*^*-ns/+*^) and homozygous *p53* mutant (*p53*^*-ns/-ns*^) (see arrows in Fig 2I and 2K). This result suggests that the loss of *p53* does not triggers programmed cell death during CNS development.

### p53 function in the larval brain is not sensitive to nutrient restriction

**Fig 3:**
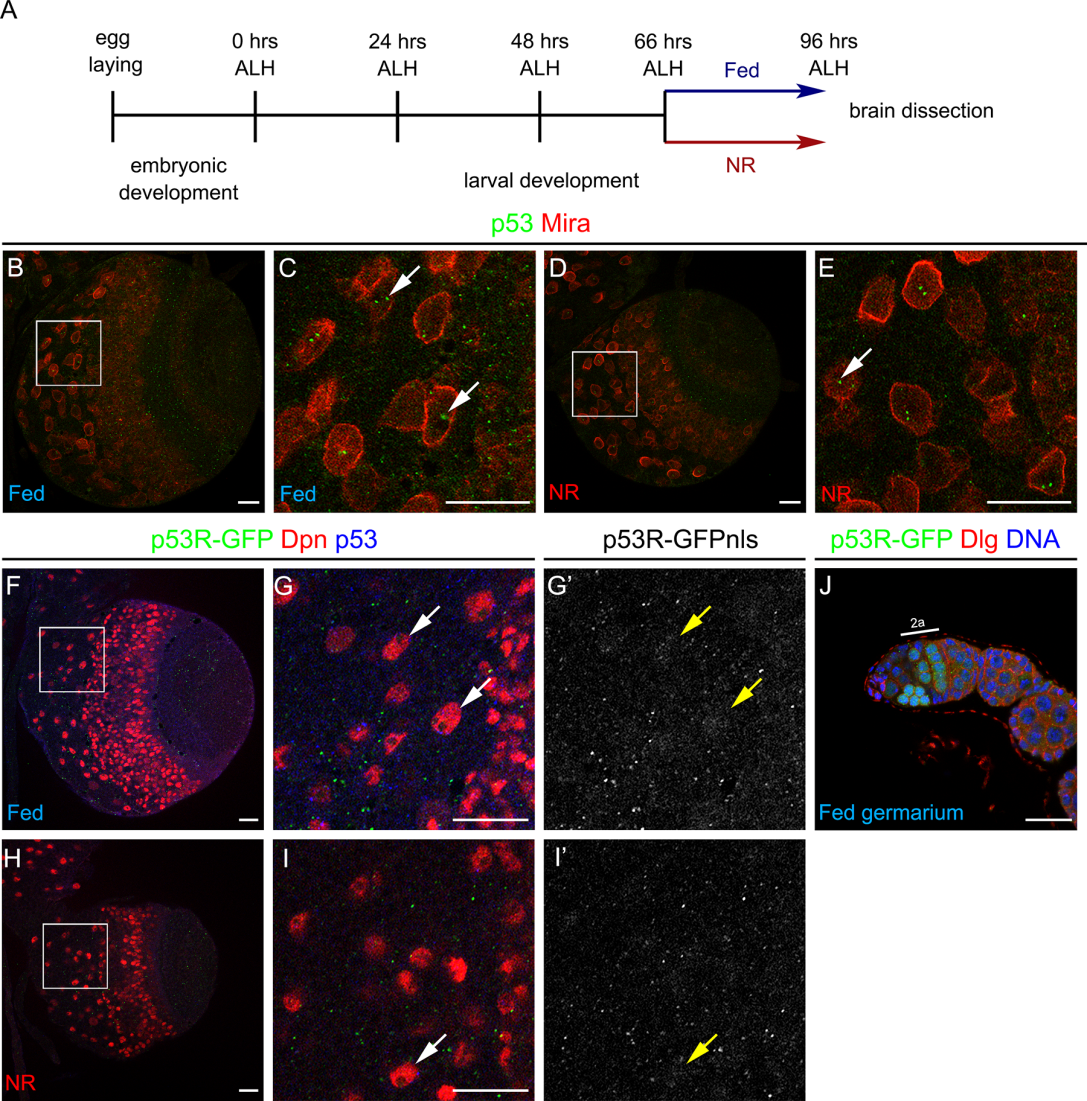
*Drosophila* p53 levels and activity in neural stem cells are independent of the nutritional condition. **(A)** Schematic representation of the nutrient restriction (NR) protocol. **(B-E)** Immunostaining against p53 (green) and Miranda (Mira, in red) of wild-type (*w*^*1118/1118*^) larval brain under **(B, C)** Fed or **(D, E)** NR conditions. **(F-I’)** Staining against GFP (green and gray), Deadpan (Dpn, neural stem cell marker in red) and p53 (blue) of larval brains of *p53* reporter (p53R-GFP) under **(F-G’)** Fed or **(H-I’)** NR conditions. Arrows point to neural stem cells. **(J)** Ovariole of *p53* reporter stock (p53R-GFP) stained for GFP (green), Dlg (red) and DNA (blue). Scale bars are 20 μm.

Given the protective role of p53 during adult nutrient restriction [5] (see S1 Fig), we assessed whether p53 has a similar function during brain development under nutritional stress. To accomplish this goal, we used a protocol in which larvae were fed until 66 hrs after larval hatching (ALH) and then transferred to a condition of full nutrient restriction (NR) until puparation (see scheme in Fig 3A). Larvae subjected to this protocol are able to puparate and generate viable adults, but the organism growth is severely reduced [32]. Importantly, the developing brain shows a preferential growth behaviour that resembles human ‘*brain sparing’* phenomenon [32, 34].

Following this protocol, we evaluated the effect of nutritional stress over p53 levels and transcriptional activity. In starved wild-type larvae, p53 levels in neural stem cells did not change compared to Fed control animals (see arrows in Fig 3B–3E). Considering that the function of p53 might be affected under NR, we used a previously described fluorescent reporter for p53 transcriptional activity (p53R-GFP) [35] to evaluate p53 activity during nutrient restriction. Interestingly, almost no fluorescence was observed in larval brain under Fed or NR conditions (Fig 3F–3I’). To confirm that the p53R-GFP stock was indeed reporting p53 endogenous transcriptional activity, we dissected adult ovarioles observing GFP expression in meiotic cells of the germarium region 2a as it has been previously reported [36] (Fig 3J). Therefore, these results suggest that p53 displays low or null transcriptional activity in neural stem cells, and this low activity does not increase after nutritional stress.

### p53 is dispensable for brain resistance to nutritional stress

**Fig 4:**
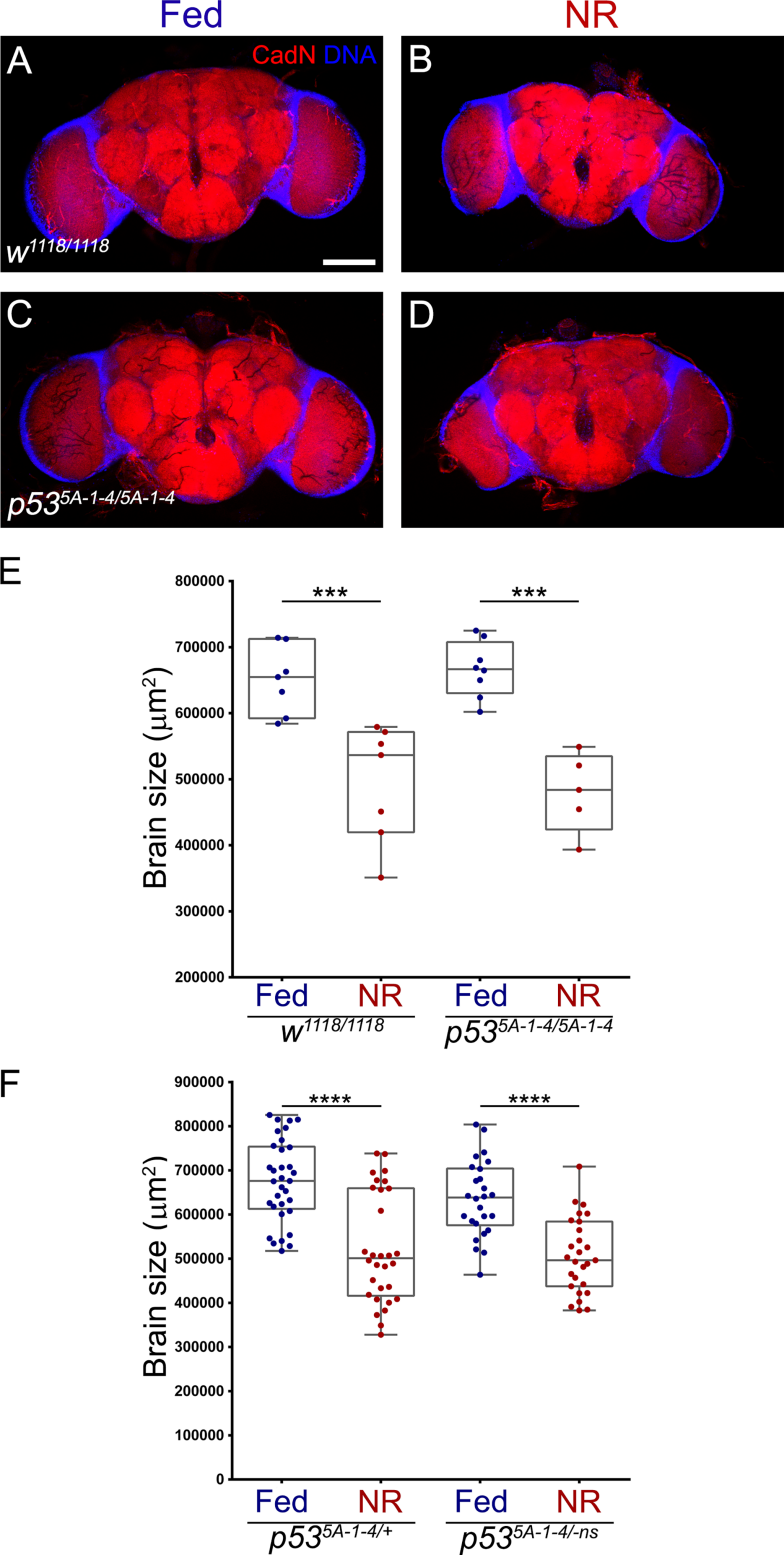
p53 does not regulate brain growth under nutrient restriction. **(A-D)** Immunofluorescence against CadN, marking the neuropil in red, and DNA (in blue) of adult female brains of **(A, B)** wild-type (*w*^*1118/1118*^) and **(C, D)** *p53* null (*p53*^*5A-1-4/5A-1-4*^) flies that were grown on **(A, C)** Fed or **(B, D)** NR conditions. Scale bar is 100 μm. **(E)** Graph showing adult female brain size quantification of wild-type (*w*^*1118/1118*^, “n” is 7 for Fed and NR) and *p53* null (*p53*^*5A-1-4/5A-1-4*^, “n” is 8 for Fed and 5 for NR) flies that were grown on Fed or NR conditions. **(F)** Graph showing adult female brain size quantification of heterozygous *p53* mutant (*p53*^*5A-1-4/+*^, “n” is 33 for Fed and 27 for NR) and *p53* null (*p53*^*5A-1-4/-ns*^, “n” is 23 for Fed and 24 for NR) flies that were grown on Fed or NR conditions.

To understand the role of p53 during brain development under nutritional stress, we assessed brain size of adult flies subjected to nutritional stress during larval development. In contrast to the results observed for adult wings, under normal Fed conditions, *p53* mutant (*p53*^*5A-1-4/5A-1-4*^) flies did not show differences in adult brain size compared to wild-type (*w*^*1118/1118*^) controls (Fig 4A and 4C). This result added to the delay on pupariation observed in *p53* mutant animals, suggests that *p53* mutant larval brain grows at slower rate than controls, and these differences may be recovered due to the longer period of larval development or during pupal stage.

Under NR conditions, we observed a significant decrease in brain size in both wild-type and *p53* mutant compared to their respective Fed conditions (Fig 4A–4E). However, we did not observe differences in brain size between wild-type and *p53* mutant animals in NR condition (Fig 4E). We confirmed that this result was independent of the genetic background by performing the experiment in heterozygous *p53* mutant (*p53*^*5A-1-4/+*^) and in a transheterozygous combination for two different *p53* null alleles (*p53*^*5A-1-4/-ns*^) (Fig 4F). These experiments show that in *p53* loss of function animals, the brain is still able to grow up to its adult size to the same extend of wild-type animals, both in normal nutrition and under nutritional stress.

### p53 does not regulate larval energy storage consumption under nutrient restriction

**Fig 5:**
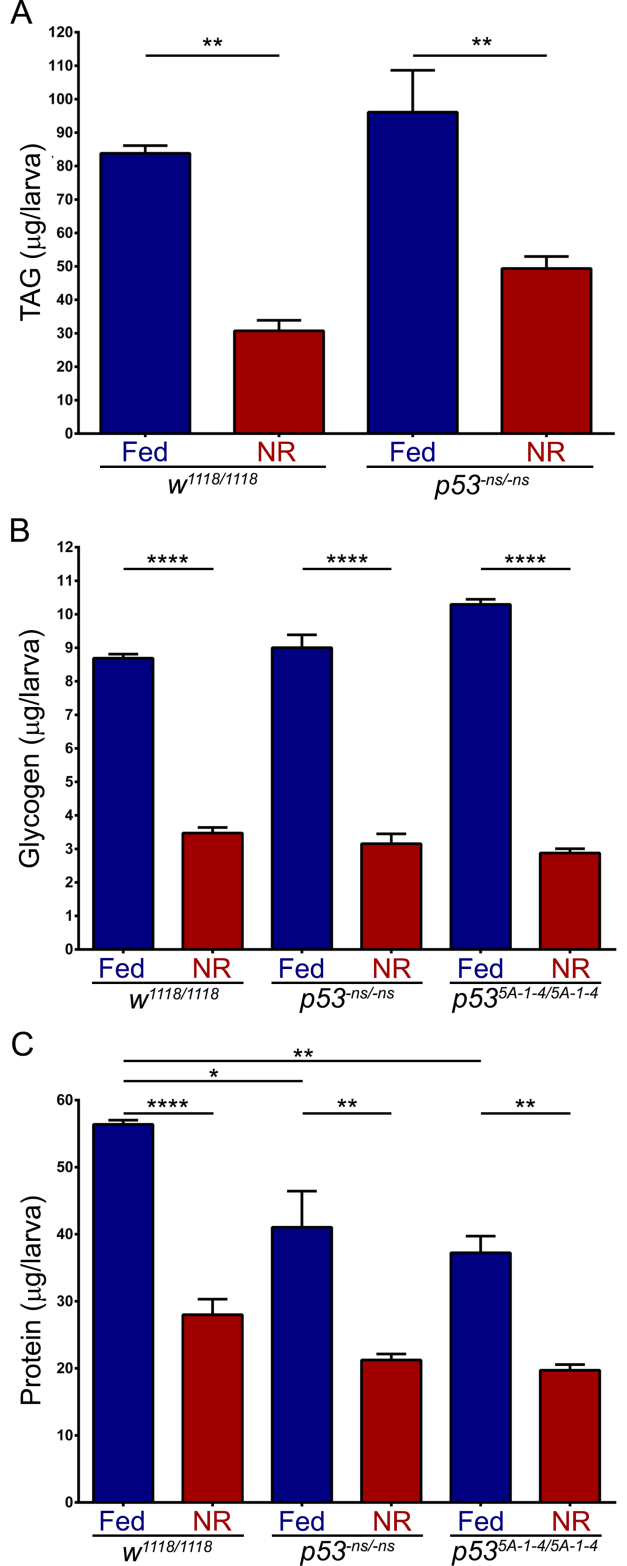
Energy storage is not affected in *p53* mutant larvae under nutrient restriction. **(A)** Graph showing total TAG quantification of wild-type (*w*^*1118*^, “n” is 3) and *p53* null (*p53*^*-ns*^, “n” is 3) larvae under Fed or NR conditions. **(B)** Graph showing total glycogen quantification of wild-type (*w*^*1118*^, “n” is 3) and *p53* null (*p53*^*-ns*^ and *p53*^*5A-1-4*^ alleles, “n” is 3) larvae under Fed or NR conditions. **(C)** Graph showing total protein quantification of wild-type (*w*^*1118*^, “n” is 3) and *p53* null (*p53*^*-ns*^ and *p53*^*5A-1-4*^ alleles, “n” is 3) larvae under Fed or NR conditions. One-way ANOVA and Tukey post-test statistical tests were used. *, ** and **** means p-value < 0.05, <0.01 and <0.001 respectively.

Previously, p53 was described to regulate energy metabolism under nutrient deprivation in adult flies. In this nutrient restriction condition, the p53R-GFP reporter is active in the adult fat body, and *p53* mutant flies are quickly depleted of triacylglycerides (TAG) and glycogen faster than wild-type flies [5]. Thus, we analysed whether this phenomenon occurs in our nutrient restriction protocol in *Drosophila* larval stage (Fig 3A). We quantified the total levels of TAG and glycogen, the main energy storages in the fat body [37], in wild-type and *p53* mutant larvae raised under Fed or NR conditions until 96 hrs ALH. Total TAG and glycogen were significantly reduced under NR in both wild-type and *p53* mutants (Fig 5A and 5B), however, no significant differences were observed between these genotypes. This suggests that p53 does not modulate energy storage metabolism during larval nutrient restriction.

In order to analyse protein levels during larval NR, we quantified total proteins, observing a significant reduction after NR for both wild-type and *p53* mutant genotypes (Fig 5C). Interestingly, in *p53* mutant larvae the total protein content was lower than in wild-type larvae under normal Fed conditions (Fig 5C), contrary to what happened to TAG and glycogen storage (Fig 5A and 5B). These results show that larval energy storages are consumed in a p53-independent manner under nutritional stress, but *p53* mutant larvae contains less total proteins that can be associated to the larval growth defects observed in *p53* mutants (Fig 1B–1D).

## Discussion

A key mechanism for organism survival is the ability to adapt to different nutritional conditions maintaining development and energy homeostasis. In this work, we analyse the function of the transcription factor p53 during larval brain development under normal and nutrient restriction conditions. We show that p53 is expressed in neural stem cells and is necessary for larval brain growth during normal development. Moreover, puparation timing and larval size are affected in *p53* mutants under normal food conditions. However, loss of *p53* function does not affect adult brain size, although reduces wing size when compared to control animals. Finally, we show that p53 is dispensable for brain resistance to developmental nutritional stress, and it does not modulate larval energy storage consumption.

### p53 controls larval growth and development in *Drosophila melanogaster*

p53 has been extensively characterised as a tumour suppressor and a stress response gene, but less is known about its normal role during development. In *Drosophila melanogaster*, null mutations for the *p53* locus produce fertile adult flies with no apparent phenotype, suggesting that p53 is not necessary during development [29, 30]. This is not completely accurate, because we detected defects in larval growth and developmental timing in *p53* mutant flies in our lab conditions. *p53* mutant larvae were smaller, had smaller brains and contained less total proteins than their wild-type counterparts (see Figs 1 and 5C). Surprisingly, in *p53* mutant adult flies, we did not find differences in brain size (see Fig 4), suggesting that this growth defect can be overcome most likely during pupal development. However, adult wings were significantly smaller than controls, confirming that these larval growth defects indeed affect adult organ size.

In the larval CNS, p53 protein was expressed in a punctate pattern in a population of neural stem cells called neuroblasts (Fig 2D and 2E). A transcriptome analysis showed that *p53* expression is enriched in this neural stem cells compared to their neuronal progeny [38]. However, an RNAi screen showed that p53 knockdown in this neural stem cell population presented no apparent phenotype [39]. Our RNAi-mediated knockdown and the expression of a dominant-negative form of *p53* affected larval brain growth, suggesting that p53 can regulate larval neurogenesis in a tissue specific manner. Moreover, in *p53* mutant mouse neural stem cells proliferation and neurogenesis are increased, but gliogenesis is reduced [40–42], showing that p53 also controls neurogenesis in vertebrates. Surprisingly, we did not observed expression of p53R-GFP reporter in *Drosophila* neural stem cells (Fig 3F–3I’), suggesting a low transcriptional activity of p53 or a function independent of transcription. Although, this reporter was previously used to detect p53 activity during nutrient restriction in adult flies, it was generated using a 150 bp enhancer from the *reaper* locus [22] and it may not completely recapitulate *p53* transcriptional activity in different cellular contexts. Alternatively, p53 may respond to a different type of stress in neural stem cells, such as oxidative stress or hypoxia during development.

How p53 regulates brain and larval growth is an interesting focus for the future. In *Drosophila*, a combination of hormones including Ecdysone, Juvenile hormone and insulin-like peptides, regulate larval growth and the transition from larval to pupal stages [1, 43]. A cross talk between p53 and the insulin pathway has been shown in vertebrate and adult flies [44, 45]. The over-expression of a short isoform of p53 triggers a hyperactivation of the insulin-like growth factor pathway in mice [46, 47]. Therefore, it is possible that during *Drosophila* larval development p53 can regulate the insulin or Ecdysone pathway promoting animal growth.

It is not clear whether p53 regulates mice growth as we observed during *Drosophila* larval development. *p53* knockout mice develop normally and are highly predisposed to generate tumours [8], however, a fraction of them die in uterus due to different defects during CNS development [11, 12]. Interestingly, loss of *p53* promotes adipogenesis and obesity in mice [48–50], suggesting that p53-mediated regulation of growth and metabolism is conserved between insects and vertebrates.

### The protective role of p53 against nutritional stress

The function of p53 has been extensively characterised in biological processes such as cancer and apoptosis, for which p53 has been commonly referred as *‘the guardian of the genome’*. In the recent years, the role of p53 regulating cellular and animal metabolism has been shown for both insects and mammals [13, 15]. In *Drosophila melanogaster*, p53 presents a protective role during adult nutritional stress, regulating the catabolic rate of energy reservoirs that permit to survive this stress condition [5]. However, we observed in our assays that energy consumption is regulated independently of p53 function in larval stages. This evidence supports a model in which p53 is dispensable for larval metabolism under nutrient restriction, but essential in adult stages.

In accordance to our results, p53 function is highly dependent on the biological context [14]. For instance, in myc-overexpressing wing disc cells, p53 normally promotes oxidative phosphorylation, but it mediates glycolysis under a cell competition context [20]. Hence, it is not surprising that p53 regulates energy storage metabolism in adult flies, but not in larvae under nutrient restriction. It may be the case that p53 has a survival role during adult nutritional stress, whereas for larval NR, in which the animal viability is not affected [32], p53 does not play a major role as a stress response gene.

How the context regulates p53 is unknown. It is plausible that p53 binds to different genomic sites in larvae and adult flies, or the presence of cofactors or binding partners may regulate p53 activity. Several reports have shown the regulation of vertebrate p53 function by different cofactors [51–55], however, it is unknown which cofactor may modulate p53 function in *Drosophila*.

## Conclusions

In this study, we describe that p53 is necessary for proper larval development in *Drosophila melanogaster*. p53 is necessary for larval growth and timing of pupariation. Adult *p53* mutant animals have smaller wings than wild-type, but normal brain size. Furthermore, p53 does not control energy consumption during larval nutrient restriction as it has been shown in adult flies subjected to a similar nutritional stress. Hence, p53 is dispensable for brain growth during nutritional stress. Thus, we conclude that during larval development p53 regulates growth but not the resistance to nutrient restriction in *Drosophila*.

## Material and methods

### Fly strains

*Drosophila melanogaster* stocks were cultured in fly food medium at 25°C. Our fly food contains the following ingredients per litter of medium: 100 g yeast, 80 g glucose, 50 g wheat flower, 11 g agar, 6 ml propionic acid, 12 ml 20% Nipagin (Methylparaben). All experiments under Fed conditions used this medium. RNAi experiments were performed at 29°C. The following fly strains were used: *w*^*1118*^ as experimental control, *p53*^*5A-1-4*^ [30], *p53*^*-ns*^ [29], p53R-GFPcyt [22], *Df(3R)ED6096* (Bloomington #8684), UAS-shp53-RNAi (p53^RNAi,^ Bloomington #41720), UAS-p53^R155H^ (p53^DN^) [24], *insc-GAL4* [56], *pros*.*MG-GAL4* (kindly gift from Chris Doe), *tub-GAL4* [57].

### Nutrient restriction protocol

The entire NR protocol was performed at 25°C. 500–800 flies were mated in an acrylic tube and eggs were collected during 3 hrs. 300 larvae were transfer immediately after hatching to a 100 mm petri dish with fly food. 66 hrs ALH, 40–50 third instar larvae were transfer to a food tube (Fed condition) or to a tube containing 1% agarose in 1x PBS (NR condition). Larval brains were dissected at 96 hr ALH, or pupae from NR condition were transfer to a food tube to dissect adult brains. For adult fly nutrient restriction, we followed Barrio *et al*. protocol [5] using 1% agar, 1% sucrose in 1x PBS.

### Immunofluorescence

Larval and adult brains were fixed in 4% formaldehyde in 1x PBS, 5 mM MgCl_2_, 0.5 mM EGTA for 20 min and stained as previously described [58] and washed with 1x PBS, 0.3% Triton X-100. Ovaries were dissected and fixed in 4% formaldehyde in 1x PBS, 0.2% Tween-20 (PBTw) for 20 min, samples were washed in PBTw. The following primary antibodies were used: rat anti-CadN 1:20 (DN-Ex #8, DSHB), mouse anti-Dlg (4F3, DSHB), guinea pig anti-Dpn 1:5000 (kind gift of A. Brand), rabbit anti-GFP 1:1000 (Life Technologies), rabbit anti-Mira 1:500 [59], mouse anti-p53 1:5 (25F4, DSHB). DNA was stained using TOPRO-3 (Molecular Probes, Invitrogen).

Alexa Fluor conjugated secondary antibodies were diluted 1:200 (Molecular Probes, Invitrogen). Primary and secondary antibodies were incubated overnight at 4°C. Brains were mounted on slides in Vectashield (Vector).

### Metabolic assays

TAG and glycogen were measured as described in [60]. Briefly, 5 larvae were homogenised in 200 μl of 1x PBS (+ 0.05% Tween 20 for TAG assay), samples heated 10 min at 70°C and store at -20°C. For TAG assay, samples were assayed using Triglyceride Reagent (SIGMA T2449) and Free Glycerol Reagent (F6428). For Glycogen assay, we used Amyloglucosidase (SIGMA A1602) and Glucose (GO) Assay Kit (SIGMA GAGO-20). Samples were incubated in duplicates 1 hr at 37°C in a 96-well plate, and absorbance at 540 nm was measured in a BioTek^TM^ Cytation 3^TM^ Cell imaging Multi-mode Reader.

### Imaging

Images were acquired using a Zeiss LSM710 or an Olympus Fluoview FV1000 confocal microscope. Images, diagrams and figures were assembled using Fiji, Adobe Photoshop CC and Illustrator CS3.

### Image processing, quantifications and statistical analysis

Larval and adult brains were quantified generating a maximum intensity projection for each confocal stack in Fiji. Later the total area of DNA (larval brains) or CadN (adult brains) signal was calculated using Bitplane Imaris 7. Pupal volume was estimated using the ellipsoid volume formula 4/3π(L/2)(d/2)^2^ (L, length; d, diameter) [61]. Wing area was calculated by measuring the total number of pixels for each wing using Adobe Photoshop CC, and normalising to the average area of the corresponding control group. Statistical analysis was performed using GraphPad Prism 5. All data and statistical analysis is available in S1 Table.

## Supporting information

S1 Fig *p53* mutant flies show less lifespan than control animals under nutrient restriction. Kaplan-Meier survival curve of male wild-type (*w*^*1118/1118*^, blue line) and *p53* mutant adult flies (*p53*^*5A-1-4/5A-1-4*^, green line) under nutrient restriction during adulthood. Median survival (dotted line) is 21 days for wild-type and 18 days *p53* mutant animals. p-value < 0.0001, Mantel-Cox test.
(TIF)

S1 Table Tables showing all data and statistical analysis for each experiment. (DOCX)
