# Supplementary figures and images for: p53 is required for brain growth but is dispensable for resistance to nutrient restriction during Drosophila larval development
Source: PLoS One. 2018 Apr 5;13(4):e0194344. doi: 10.1371/journal.pone.0194344 (PMC5886404; doi:10.1371/journal.pone.0194344)

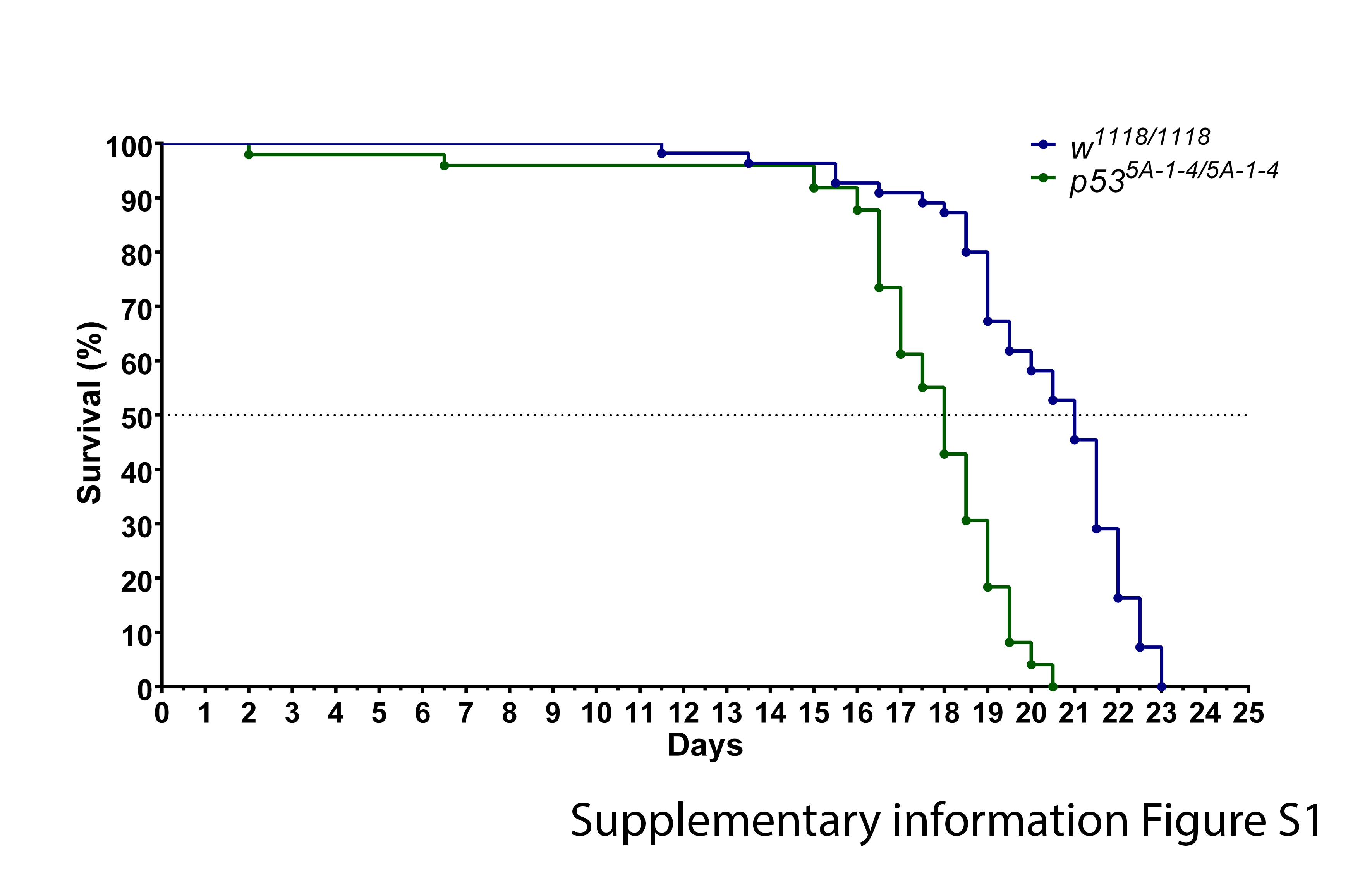

Supplement: S1 Fig — Kaplan-Meier survival curve of male wild-type (w1118/1118, blue line) and p53 mutant adult flies (p535A-1-4/5A-1-4, green line) under nutrient restriction during adulthood. Median survival (dotted line) is 21 days for wild-type and 18 days p53 mutant animals. p-value < 0.0001, Mantel-Cox test. (TIF) [file pone.0194344.s001.tif]
