# Supplementary material for: p53 is required for brain growth but is dispensable for resistance to nutrient restriction during Drosophila larval development
Source: PLoS One. 2018 Apr 5;13(4):e0194344. doi: 10.1371/journal.pone.0194344 (PMC5886404; doi:10.1371/journal.pone.0194344)
Supplement: S1 Table — (DOCX) [file pone.0194344.s002.docx]

**Supporting information**

**Data for Figure 1A. Percentage of pupariation.**

| **Time (hrs)** | ***w^1118^*** | | | | | ***p53^5A-1-4^*** | | | | |
| --- | --- | --- | --- | --- | --- | --- | --- | --- | --- | --- |
|  | **1** | **2** | **3** | **4** | **5** | **1** | **2** | **3** | **4** | **5** |
| **96** | 0.00 | 0.00 | 0.00 | 0.00 | 0.00 | 0.00 | 0.00 | 0.00 | 0.00 | 0.00 |
| **98** | 0.00 | 0.00 | 0.00 | 0.00 | 0.00 | 0.00 | 0.00 | 0.00 | 0.00 | 0.00 |
| **100** | 0.00 | 0.00 | 0.00 | 0.00 | 0.00 | 0.00 | 2.50 | 0.00 | 0.00 | 0.00 |
| **101** | 0.00 | 0.00 | 0.00 | 0.00 | 0.00 | 0.00 | 2.50 | 0.00 | 0.00 | 0.00 |
| **102** | 0.00 | 0.00 | 0.00 | 2.70 | 0.00 | 5.00 | 2.50 | 0.00 | 0.00 | 0.00 |
| **103** | 0.00 | 0.00 | 0.00 | 5.41 | 0.00 | 5.00 | 2.50 | 0.00 | 0.00 | 0.00 |
| **104** | 2.56 | 2.56 | 2.70 | 5.41 | 5.13 | 5.00 | 5.00 | 0.00 | 0.00 | 0.00 |
| **105** | 10.26 | 7.69 | 8.11 | 8.11 | 15.38 | 5.00 | 7.50 | 2.70 | 0.00 | 0.00 |
| **106** | 15.38 | 10.26 | 13.51 | 13.51 | 23.08 | 7.50 | 7.50 | 2.70 | 0.00 | 0.00 |
| **107** | 25.64 | 10.26 | 16.22 | 18.92 | 28.21 | 10.00 | 12.50 | 2.70 | 2.50 | 2.56 |
| **108** | 33.33 | 20.51 | 24.32 | 29.73 | 33.33 | 15.00 | 12.50 | 8.11 | 2.50 | 7.69 |
| **111** | 51.28 | 33.33 | 45.95 | 56.76 | 61.54 | 15.00 | 17.50 | 13.51 | 10.00 | 30.77 |
| **114** | 69.23 | 53.85 | 67.57 | 62.16 | 71.79 | 20.00 | 37.50 | 37.84 | 32.50 | 38.46 |
| **115** | 74.36 | 56.41 | 72.97 | 67.57 | 76.92 | 32.50 | 40.00 | 48.65 | 32.50 | 46.15 |
| **116** | 82.05 | 58.97 | 86.49 | 75.68 | 76.92 | 40.00 | 40.00 | 64.86 | 50.00 | 61.54 |
| **117** | 84.62 | 74.36 | 97.30 | 83.78 | 76.92 | 50.00 | 50.00 | 70.27 | 55.00 | 64.10 |
| **118** | 89.74 | 79.49 | 100.00 | 86.49 | 84.62 | 55.00 | 57.50 | 75.68 | 62.50 | 74.36 |
| **119** | 89.74 | 82.05 | 100.00 | 91.89 | 87.18 | 65.00 | 62.50 | 81.08 | 72.50 | 76.92 |
| **121** | 92.31 | 92.31 | 100.00 | 94.59 | 94.87 | 80.00 | 87.50 | 89.19 | 85.00 | 84.62 |
| **124** | 92.31 | 97.44 | 100.00 | 94.59 | 97.44 | 90.00 | 92.50 | 100.00 | 100.00 | 100.00 |
| **126** | 92.31 | 97.44 | 100.00 | 94.59 | 100.00 | 95.00 | 95.00 | 100.00 | 100.00 | 100.00 |
| **140** | 100.00 | 100.00 | 100.00 | 100.00 | 100.00 | 100.00 | 100.00 | 100.00 | 100.00 | 100.00 |

**Data for Figure 1C**. Pupal volume (mm^3^). Statistical analysis: Two-way ANOVA with Bonferroni’s multiple comparisons.

|  | **Female** | | **Male** | |
| --- | --- | --- | --- | --- |
| **Pupa** | ***w^1118^*** | ***p53^5A-1-4^*** | ***w^1118^*** | ***p53^5A-1-4^*** |
| **1** | 1.904983 | 1.594245 | 2.166756 | 1.438550 |
| **2** | 1.966256 | 1.583135 | 1.565196 | 1.179371 |
| **3** | 2.039824 | 1.536201 | 1.637042 | 1.313683 |
| **4** | 1.916529 | 1.453278 | 1.554933 | 1.191023 |
| **5** | 1.947325 | 1.645213 | 1.706932 | 1.132387 |
| **6** | 2.147750 | 1.568719 | 1.523679 | 1.245319 |
| **7** | 2.009287 | 1.575663 | 1.570328 | 1.258952 |
| **8** | 1.984240 | 1.413049 | 1.555088 | 1.111181 |
| **9** | 2.050181 | 1.714485 | 1.677147 | 1.256977 |
| **10** | 1.870844 | 1.392773 | 1.528707 | 1.346311 |
| **11** | 2.108877 | 1.934693 | 2.033710 | 1.278754 |
| **12** | 2.109736 | 1.623361 | 1.699627 | 1.359725 |
| **13** | 1.990965 | 2.016383 | 1.139385 | 1.272293 |
| **14** | 1.887665 | 1.413917 | 1.634417 | 1.508747 |
| **15** | 1.929675 | 1.293230 | 1.649336 | 1.290799 |
| **16** | 1.864575 | 1.236309 | 1.538765 | 0.9605734 |
| **17** | 1.935558 | 1.528908 | 1.527228 | 1.236455 |
| **18** | 1.947325 | 1.230654 | 1.333954 | 1.066399 |
| **19** | 2.193429 | 1.290799 | 1.666942 | 1.341485 |
| **20** | 1.929675 | 1.484862 | 1.263040 | 1.313931 |
| **21** | 1.923786 | 1.354799 | 1.805329 | 1.244534 |
| **22** | 2.122465 | 1.249160 | 1.897187 | 1.196004 |
| **23** | 1.858802 | 1.804399 | 1.443474 | 1.254182 |
| **24** | 2.014213 | 1.397963 | 1.529274 | 1.285330 |
| **25** | 1.904983 | 1.649793 | 1.590676 | 1.228625 |
| **26** | 2.090215 | 1.408020 | 1.394016 | 1.327009 |
| **27** | 2.046669 | 1.195552 | 1.462561 | 1.223491 |
| **26** | 1.881035 | 1.558623 | 1.453330 | 1.276919 |
| **29** | 1.179040 | 1.813855 | 1.560065 | 1.257417 |
| **30** | 1.899211 | 1.762437 | 1.585724 | 1.235738 |
| **Avg** | 1.955 | 1.524 | 1.590 | 1.254 |
| **SD** | 0.1732 | 0.2107 | 0.2043 | 0.1041 |
| **SEM** | 0.03163 | 0.03847 | 0.03730 | 0.01901 |
| **p-value** | <0.0001 | | <0.0001 | |

**Data for Figure 1D**. Pupal volume (mm^3^). Statistical analysis: Unpaired t-test.

| **Pupa** | **Ctrl** | **p53^RNAi^** |
| --- | --- | --- |
| **1** | 1.623734 | 1.858352 |
| **2** | 1.947134 | 1.900314 |
| **3** | 1.812620 | 1.766439 |
| **4** | 2.238743 | 1.841428 |
| **5** | 1.947134 | 1.812012 |
| **6** | 1.906142 | 1.789530 |
| **7** | 1.960429 | 1.789530 |
| **8** | 2.046381 | 1.823565 |
| **9** | 1.930288 | 1.818393 |
| **10** | 1.870341 | 1.683284 |
| **11** | 1.978239 | 1.928472 |
| **12** | 1.953355 | 1.912304 |
| **13** | 2.119409 | 1.762437 |
| **14** | 2.206325 | 1.744226 |
| **15** | 1.710300 | 2.108335 |
| **16** | 1.806848 | 1.858352 |
| **17** | 1.642341 | 1.754894 |
| **18** | 1.954322 | 1.800912 |
| **19** | 1.922251 | 1.666464 |
| **20** | 1.882610 | 1.759064 |
| **21** | 2.106037 | 1.601119 |
| **22** | 2.027375 | 1.766932 |
| **23** | 2.092666 | 1.894376 |
| **24** | 1.911571 | 1.517375 |
| **25** | 2.006342 | 1.749943 |
| **26** | 2.046381 | 1.772000 |
| **27** | 1.948215 | 1.623734 |
| **26** | 1.957683 | 1.617914 |
| **29** | 1.976690 | 1.788479 |
| **30** | 1.912304 | 1.972250 |
| **31** | 1.954322 | 1.812620 |
| **32** | 1.829661 | 1.727290 |
| **33** | 1.888325 | 1.800246 |
| **34** | 1.912304 | 1.661494 |
| **35** | 1.870341 | 1.948215 |
| **36** | 1.947134 | 1.618393 |
| **37** | - | 1.629076 |
| **38** | - | 1.829661 |
| **Avg** | 1.940 | 1.782 |
| **SD** | 0.1298 | 0.1173 |
| **SEM** | 0.02163 | 0.01902 |
| **p-value** | <0.0001 | |

**Data for Figure 1F**. Wing area (µm^2^). Statistical analysis: Two-way ANOVA with Bonferroni’s multiple comparisons.

|  | **Female** | | **Male** | |
| --- | --- | --- | --- | --- |
| **Wing** | ***w^1118^*** | ***p53^5A-1-4^*** | ***w^1118^*** | ***p53^5A-1-4^*** |
| **1** | 1.02 | 0.91 | 0.79 | 0.59 |
| **2** | 1.01 | 0.88 | 0.77 | 0.71 |
| **3** | 1.01 | 0.92 | 0.77 | 0.66 |
| **4** | 1.03 | 0.88 | 0.75 | 0.64 |
| **5** | 1.02 | 0.83 | 0.82 | 0.69 |
| **6** | 0.99 | 0.86 | 0.79 | 0.66 |
| **7** | 1.04 | 0.72 | 0.76 | 0.65 |
| **8** | 1.04 | 0.87 | 0.74 | 0.69 |
| **9** | 1.00 | 0.83 | 0.76 | 0.66 |
| **10** | 1.00 | 0.87 | 0.82 | 0.68 |
| **11** | 0.98 | 0.81 | 0.80 | 0.67 |
| **12** | 0.99 | 0.81 | 0.80 | 0.64 |
| **13** | 1.01 | 0.90 | 0.78 | 0.64 |
| **14** | 0.99 | 0.86 | 0.79 | 0.67 |
| **15** | 0.95 | 0.94 | 0.77 | 0.66 |
| **16** | 1.00 | 0.88 | 0.78 | 0.65 |
| **17** | 1.00 | 0.85 | 0.79 | 0.59 |
| **18** | 0.95 | 0.85 | 0.78 | 0.66 |
| **19** | 0.98 | 0.85 | 0.78 | 0.66 |
| **20** | - | 0.87 | 0.79 | 0.57 |
| **21** | - | 0.85 | 0.81 | - |
| **22** | - | - | 0.78 | - |
| **23** | - | - | 0.78 | - |
| **Avg** | 1.001 | 0.8590 | 0.7826 | 0.6520 |
| **SD** | 0.02505 | 0.04603 | 0.02005 | 0.03473 |
| **SEM** | 0.005747 | 0.01005 | 0.004181 | 0.007766 |
| **p-value** | <0.0001 | | <0.0001 | |

**Data for Figure 1G**. Wing area (µm^2^). Statistical analysis: One-way ANOVA with Tukey’s post test.

| **Wing** | ***w^1118^*** | ***p53^5A-1-4/+^*** | ***p53^5A-1-4/5A-1-4^*** | ***p53^5A-1-4^/*** *Df(3R)ED6096* |
| --- | --- | --- | --- | --- |
| **1** | 1.05 | 1.03 | 0.99 | 1.02 |
| **2** | 1.02 | 1.04 | 0.83 | 1.00 |
| **3** | 1.01 | 1.03 | 0.89 | 1.00 |
| **4** | 0.99 | 1.04 | 0.90 | 0.95 |
| **5** | 1.01 | 1.04 | 0.93 | 0.97 |
| **6** | 0.97 | 1.00 | 0.94 | 0.98 |
| **7** | 1.03 | 1.04 | 0.94 | 0.96 |
| **8** | 0.96 | 1.07 | 0.80 | 0.94 |
| **9** | 0.96 | 1.00 | 0.96 | 1.00 |
| **10** | 1.00 | 1.04 | 0.90 | 0.99 |
| **11** | 0.98 | 1.02 | 0.99 | 0.97 |
| **12** | 0.99 | 1.01 | 0.90 | 1.00 |
| **13** | 1.00 | 1.05 | 0.93 | 0.98 |
| **14** | 1.05 | 0.99 | 0.93 | 0.99 |
| **15** | 1.00 | 1.05 | 0.91 | 1.00 |
| **16** | 1.01 | 1.07 | 0.90 | 1.00 |
| **17** | 0.97 | 1.04 | 0.93 | 1.04 |
| **18** | 0.99 | 1.00 | 0.93 | 0.96 |
| **19** | 0.99 | 1.03 | 0.95 | 0.97 |
| **20** | 0.98 | 1.02 | 0.93 | 0.95 |
| **21** | 1.01 | 1.00 | 0.96 | 0.99 |
| **22** | 0.99 | 1.02 | 0.83 | 0.99 |
| **23** | 1.02 | 1.03 | 0.94 | 0.99 |
| **24** | 1.01 | 1.00 | 0.84 | 0.99 |
| **25** | 1.01 | 1.07 | 0.94 | 1.01 |
| **26** | 0.99 | 1.01 | 0.88 | 0.96 |
| **27** | 1.02 | 1.03 | - | 0.94 |
| **28** | 1.01 | 0.94 | - | 0.97 |
| **29** | 0.99 | 1.02 | - | 1.00 |
| **30** | - | - | - | 0.96 |
| **31** | - | - | - | 0.92 |
| **32** | - | - | - | 0.94 |
| **Avg** | 1.000 | 1.025 | 0.9142 | 0.9791 |
| **SD** | 0.02260 | 0.02734 | 0.04751 | 0.02644 |
| **SEM** | 0.004196 | 0.005076 | 0.009318 | 0.004674 |
| **p-value** | Compare to | 0.0187 | <0.0001 | 0.0498 |
| **p-value** | X | Compare to | <0.0001 | <0.0001 |
| **p-value** | X | X | Compare to | <0.0001 |

**Data for Figure 1H**. Wing area (µm^2^). Statistical analysis: Two-way ANOVA with Bonferroni’s multiple comparisons.

|  | **Female** | | **Male** | |
| --- | --- | --- | --- | --- |
| **Wing** | ***w^1118^*** | ***p53^5A-1-4^*** | ***w^1118^*** | ***p53^5A-1-4^*** |
| **1** | 1.02 | 1.02 | 0.74 | 0.73 |
| **2** | 1.01 | 0.92 | 0.76 | 0.73 |
| **3** | 0.95 | 0.97 | 0.76 | 0.76 |
| **4** | 0.96 | 1.04 | 0.77 | 0.79 |
| **5** | 1.01 | 0.90 | 0.78 | 0.72 |
| **6** | 0.99 | 0.92 | 0.76 | 0.73 |
| **7** | 1.01 | 0.96 | 0.74 | 0.73 |
| **8** | 1.01 | 0.98 | 0.74 | 0.76 |
| **9** | 1.00 | 0.96 | 0.75 | 0.70 |
| **10** | 1.02 | 0.96 | 0.77 | 0.75 |
| **11** | 0.99 | 0.98 | 0.78 | 0.74 |
| **12** | 0.99 | 0.97 | 0.76 | 0.76 |
| **13** | 1.03 | 0.98 | 0.72 | 0.73 |
| **14** | 0.97 | 0.95 | 0.74 | 0.73 |
| **15** | 0.98 | 1.00 | 0.75 | 0.73 |
| **16** | 1.04 | 0.97 | 0.75 | 0.74 |
| **17** | 0.99 | 0.95 | 0.77 | 0.73 |
| **18** | 0.98 | 0.97 | 0.77 | 0.74 |
| **19** | 1.03 | 0.99 | 0.77 | 0.75 |
| **20** | 1.01 | 0.94 | 0.77 | 0.76 |
| **21** | 1.00 | 0.92 | 0.76 | 0.77 |
| **22** | - | 0.95 | 0.75 | 0.79 |
| **23** | - | 0.98 | 0.78 | 0.78 |
| **24** | - | - | 0.77 | - |
| **Avg** | 0.9995 | 0.9643 | 0.7588 | 0.7457 |
| **SD** | 0.02334 | 0.03245 | 0.01541 | 0.02273 |
| **SEM** | 0.005093 | 0.006766 | 0.003146 | 0.004739 |
| **p-value** | <0.0001 | | 0.1337 | |

**Data for Figure 2A**. Larval brain size (µm^2^). Statistical analysis: Unpaired t-test.

| **Brain** | ***w^1118^*** | ***p53^5A-1-4^*** |
| --- | --- | --- |
| **1** | 867336.600 | 543941.600 |
| **2** | 824047.000 | 521085.300 |
| **3** | 793689.000 | 559121.600 |
| **4** | 729713.200 | 629351.800 |
| **5** | 927389.100 | 526291.500 |
| **6** | 860282.900 | 605195.400 |
| **7** | 807131.300 | 620109.800 |
| **8** | 976231.400 | 590375.300 |
| **9** | 736550.400 | 607616.800 |
| **10** | 837749.900 | 514522.300 |
| **Avg** | 836012 | 571761 |
| **SD** | 77133 | 43779 |
| **SEM** | 24392 | 13844 |
| **p-value** | <0.0001 | |

**Data for Figure 2B**. Larval brain size (µm^2^). Statistical analysis: Unpaired t-test.

| **Brain** | ***p53^-ns/+^*** | ***p53^-ns/-ns^*** |
| --- | --- | --- |
| **1** | 756118.100 | 799935.400 |
| **2** | 834999.400 | 667390.800 |
| **3** | 900790.900 | 716144.900 |
| **4** | 920957.300 | 831894.000 |
| **5** | 821206.700 | 724383.800 |
| **6** | 971234.400 | 690608.500 |
| **7** | 894971.700 | 761809.000 |
| **8** | 911767.800 | 600173.000 |
| **9** | 930664.500 | 675400.000 |
| **10** | 917017.100 | 724905.700 |
| **11** | 882324.300 | 741853.800 |
| **12** | 887689.600 | 674973.500 |
| **13** | 922684.800 | 686122.300 |
| **14** | 927890.800 | 578551.300 |
| **15** | 826889.900 | - |
| **Avg** | 887147 | 705296 |
| **SD** | 55205 | 68685 |
| **SEM** | 14254 | 18357 |
| **p-value** | <0.0001 | |

**Data for Figure 2C**. Larval brain size (µm^2^). Statistical analysis: Unpaired t-test.

| **Brain** | ***p53^5A-1-4/+^*** | ***p53^5A-1-4/-ns^*** |
| --- | --- | --- |
| **1** | 865571.600 | 692507.700 |
| **2** | 725345.400 | 565435.100 |
| **3** | 758446.400 | 656181.800 |
| **4** | 781705.300 | 572091.900 |
| **5** | 736187.400 | 742485.400 |
| **6** | 744444.300 | 648245.500 |
| **7** | 840975.100 | 638488.100 |
| **8** | 704903.200 | 695240.400 |
| **9** | 881874.300 | 635292.200 |
| **10** | 563920.400 | 619349.800 |
| **11** | 590603.700 | 663553.800 |
| **12** | - | 753662.500 |
| **Avg** | 744907 | 656878 |
| **SD** | 101347 | 58247 |
| **SEM** | 30557 | 16814 |
| **p-value** | 0.0174 | |

**Data for Figure 2L**. Larval brain size (µm^2^). Statistical analysis: Unpaired t-test.

| **Brain** | **Ctrl** | **p53^DN^** |
| --- | --- | --- |
| **1** | 848783.600 | 614593.100 |
| **2** | 855673.600 | 900738.100 |
| **3** | 835708.400 | 580385.400 |
| **4** | 762371.400 | 698372.100 |
| **5** | 794518.400 | 884169.500 |
| **6** | 861294.900 | 835619.900 |
| **7** | 908474.900 | 835467.700 |
| **8** | 844597.200 | 728203.600 |
| **9** | 791383.900 | 667151.400 |
| **10** | 884020.300 | 811561.200 |
| **Avg** | 838683 | 755626 |
| **SD** | 44576 | 113500 |
| **SEM** | 14096 | 35892 |
| **p-value** | 0.0450 | |

**Data for Figure 2M**. Larval brain size (µm^2^). Statistical analysis: Unpaired t-test.

| **Brain** | **Ctrl** | **p53^RNAi^** |
| --- | --- | --- |
| **1** | 701888.900 | 639942.100 |
| **2** | 830401.700 | 422754.200 |
| **3** | 748186.300 | 489316.900 |
| **4** | 617620.300 | 545870.300 |
| **5** | 806207.400 | 539953.800 |
| **6** | 838534.400 | 671720.500 |
| **7** | 826109.200 | 561500.400 |
| **8** | 885425.100 | 578724.100 |
| **9** | 697670.100 | 528862.200 |
| **10** | 769703.800 | 441508.600 |
| **Avg** | 772175 | 542015 |
| **SD** | 81409 | 78482 |
| **SEM** | 25744 | 24818 |
| **p-value** | <0.0001 | |

**Data for Figure 4E**. Adult brain size (µm^2^). Statistical analysis: One-way ANOVA with Tukey’s post test.

| **Brain** | ***w^1118/1118^* Fed** | ***w^1118/1118^* NR** | ***p53^5A-1-4/5A-1-4^* Fed** | ***p53^5A-1-4/5A-1-4^* NR** |
| --- | --- | --- | --- | --- |
| **1** | 714255.900 | 351148.600 | 725079.300 | 549082.100 |
| **2** | 712522.800 | 451026.900 | 716857.300 | 393463.700 |
| **3** | 654764.700 | 419797.500 | 664710.700 | 483925.300 |
| **4** | 662926.500 | 571630.100 | 650035.200 | 454607.000 |
| **5** | 584113.100 | 536583.200 | 680356.600 | 520768.000 |
| **6** | 592258.100 | 579277.700 | 623835.200 | - |
| **7** | 632416.900 | 553513.800 | 602068.000 | - |
| **8** | - | - | 668707.400 | - |
| **Avg** | 650465 | 494711 | 666456 | 480369 |
| **SD** | 51978 | 87948 | 42106 | 60379 |
| **SEM** | 19646 | 33241 | 14887 | 27002 |
| **p-value** | Compare to | 0.0006 | 0.9594 | 0.0006 |
| **p-value** | X | Compare to | 0.0001 | 0.9790 |
| **p-value** | X | X | Compare to | 0.0001 |

**Data for Figure 4F**. Adult brain size (µm^2^). Statistical analysis: One-way ANOVA with Tukey’s post test.

| **Brain** | ***p53^5A-1-4/+^* Fed** | ***p53^5A-1-4/+^* NR** | ***p53^5A-1-4/-ns^* Fed** | ***p53^5A-1-4/-ns^* NR** |
| --- | --- | --- | --- | --- |
| **1** | 746635.300 | 737129.400 | 803835.800 | 584106.800 |
| **2** | 796259.600 | 608559.900 | 719652.200 | 564905.300 |
| **3** | 814971.900 | 659074.800 | 707704.400 | 622582.300 |
| **4** | 814971.900 | 698833.800 | 792499.200 | 422285.700 |
| **5** | 812650.100 | 695164.800 | 740757.000 | 488495.400 |
| **6** | 825430.800 | 675674.300 | 596176.600 | 602120.300 |
| **7** | 752331.200 | 738219.300 | 564373.500 | 602574.900 |
| **8** | 625942.400 | 451599.500 | 703171.500 | 525712.100 |
| **9** | 607921.800 | 495932.200 | 521064.500 | 514670.900 |
| **10** | 642537.300 | 400525.300 | 659375.800 | 541164.800 |
| **11** | 706199.300 | 372402.900 | 513809.900 | 442072.300 |
| **12** | 755528.600 | 418302.900 | 644401.300 | 586832.100 |
| **13** | 653100.900 | 408561.200 | 640891.500 | 496458.800 |
| **14** | 676077.400 | 433181.900 | 642375.800 | 628812.600 |
| **15** | 617953.400 | 407806.800 | 635849.200 | 390976.000 |
| **16** | 706640.600 | 348806.500 | 541631.300 | 382981.500 |
| **17** | 694263.900 | 511552.100 | 596743.800 | 421680.300 |
| **18** | 682599.400 | 507234.700 | 585043.000 | 493245.500 |
| **19** | 707876.800 | 485897.900 | 680449.900 | 456838.100 |
| **20** | 600870.600 | 506080.500 | 556556.800 | 465477.800 |
| **21** | 632596.800 | 382563.000 | 463599.600 | 402412.400 |
| **22** | 534638.200 | 327876.300 | 615601.500 | 527643.100 |
| **23** | 553418.300 | 482582.800 | 596450.800 | 384824.700 |
| **24** | 623650.100 | 489149.400 | - | 437510.500 |
| **25** | 517601.600 | 436336.400 | - | - |
| **26** | 675421.500 | 507625.700 | - | - |
| **27** | 545923.400 | 515809.700 | - | - |
| **28** | 540118.400 | - | - | - |
| **29** | 699457.000 | - | - | - |
| **30** | 528677.300 | - | - | - |
| **31** | 746635.300 | - | - | - |
| **32** | 796259.600 | - | - | - |
| **33** | 814971.900 | - | - | - |
| **Avg** | 676106 | 523239 | 634994 | 506652 |
| **SD** | 92895 | 124871 | 85515 | 85141 |
| **SEM** | 16171 | 22798 | 16771 | 16385 |
| **p-value** | Compare to | <0.0001 | 0.3929 | <0.0001 |
| **p-value** | X | Compare to | 0.0003 | 0.9218 |
| **p-value** | X | X | Compare to | <0.0001 |

**Data for Figure 5A**. Triacylgliceride (µg/larva). Statistical analysis: One-way ANOVA with Tukey’s post test.

| **Brain** | ***w^1118/1118^* Fed** | ***w^1118/1118^* NR** | ***p53^-ns/-ns^* Fed** | ***p53^-ns/-ns^* NR** |
| --- | --- | --- | --- | --- |
| **1** | 79.620850 | 35.854110 | 86.791670 | 51.926640 |
| **2** | 84.318980 | 31.403260 | 80.609930 | 53.904800 |
| **3** | 87.533490 | 24.974240 | 120.914900 | 42.283120 |
| **Avg** | 83.82 | 30.74 | 96.11 | 49.37 |
| **SD** | 3.979 | 5.470 | 21.71 | 6.218 |
| **SEM** | 2.298 | 3.158 | 12.53 | 3.590 |
| **p-value** | Compare to | 0.0025 | 0.6010 | 0.0295 |
| **p-value** | X | Compare to | 0.0006 | 0.2867 |
| **p-value** | X | X | Compare to | 0.0055 |

**Data for Figure 5B**. Glycogen (µg/larva). Statistical analysis: One-way ANOVA with Tukey’s post test.

| **Brain** | ***w^1118/1118^* Fed** | ***w^1118/1118^* NR** | ***p53^-ns/-ns^* Fed** | ***p53^-ns/-ns^* NR** | ***p53^5A-1-4/5A-1-4^* Fed** | ***p53^5A-1-4/5A-1-4^* NR** |
| --- | --- | --- | --- | --- | --- | --- |
| **1** | 8.739 | 3.1500 | 8.328 | 2.561 | 10.006 | 3.122 |
| **2** | 8.872 | 3.7170 | 9.656 | 3.417 | 10.511 | 2.683 |
| **3** | 8.456 | 3.5500 | 9.028 | 3.483 | 10.372 | 2.822 |
| **Avg** | 8.689 | 3.472 | 9.004 | 3.154 | 10.30 | 2.876 |
| **SD** | 0.2125 | 0.2914 | 0.6643 | 0.5143 | 0.2609 | 0.2244 |
| **SEM** | 0.1227 | 0.1682 | 0.3835 | 0.2969 | 0.1506 | 0.1295 |
| **p-value** | Compare to | <0.0001 | 0.9198 | < 0.0001 | 0.0036 | <0.0001 |
| **p-value** | X | Compare to | <0.0001 | 0.9163 | < 0.0001 | 0.4824 |
| **p-value** | X | X | Compare to | < 0.0001 | 0.0179 | < 0.0001 |
| **p-value** | X | X | X | Compare to | < 0.0001 | 0.9507 |
| **p-value** | X | X | X | X | Compare to | < 0.0001 |

**Data for Figure 5C**. Protein (µg/larva). Statistical analysis: One-way ANOVA with Tukey’s post test.

| **Brain** | ***w^1118/1118^* Fed** | ***w^1118/1118^* NR** | ***p53^-ns/-ns^* Fed** | ***p53^-ns/-ns^* NR** | ***p53^5A-1-4/5A-1-4^* Fed** | ***p53^5A-1-4/5A-1-4^* NR** |
| --- | --- | --- | --- | --- | --- | --- |
| **1** | 56.857140 | 28.571430 | 43.142860 | 19.714280 | 41.714290 | 21.428570 |
| **2** | 57.142860 | 31.714280 | 49.142860 | 21.142860 | 33.142860 | 18.857140 |
| **3** | 55.142860 | 23.714280 | 30.857140 | 22.857140 | 36.857140 | 18.857140 |
| **Avg** | 56.38 | 28.00 | 41.05 | 21.24 | 37.24 | 19.71 |
| **SD** | 1.082 | 4.030 | 9.321 | 1.574 | 4.298 | 1.485 |
| **SEM** | 0.6245 | 2.327 | 5.382 | 0.9085 | 2.482 | 0.8571 |
| **p-value** | Compare to | <0.0001 | 0.0150 | <0.0001 | 0.0028 | <0.0001 |
| **p-value** | X | Compare to | 0.0417 | 0.5018 | 0.2122 | 0.3041 |
| **p-value** | X | X | Compare to | 0.0021 | 0.9051 | 0.0011 |
| **p-value** | X | X | X | Compare to | 0.0111 | 0.9982 |
| **p-value** | X | X | X | X | Compare to | 0.0057 |
